# Antibody-Conjugated Electrospun Nanofibers for Electrochemical Detection of Methamphetamine

**DOI:** 10.1021/acsami.3c02266

**Published:** 2023-05-15

**Authors:** Gozde Atik, Nur Melis Kilic, Nesrin Horzum, Dilek Odaci, Suna Timur

**Affiliations:** † Department of Biochemistry, Faculty of Science, Ege University, Bornova, 35100 Izmir, Turkey; ‡ Department of Engineering Sciences and Biocomposite Engineering Graduate Program, İzmir Katip Çelebi University, 35620 Izmir, Turkey; § Central Research Test and Analysis Laboratory Application and Research Center, Ege University, Bornova, 35100 Izmir, Turkey

**Keywords:** nanobiotechnology, nanotechnology, nanofibers, immunosensor, metamphetamine

## Abstract

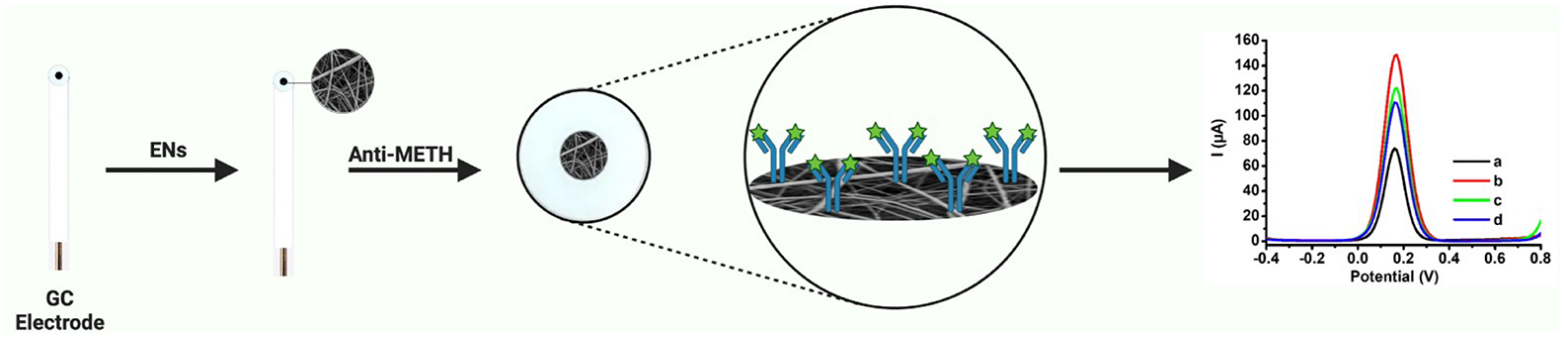

Multifunctional electrospun nanofibers (ENs) with improved properties have increased attention nowadays. Their insoluble forms in water with decreased hydrophobicity are desired for the immobilization of biological molecules. Also, the addition of functional groups on the backbone provides the conjugation of biomolecules onto the surface of ENs via covalent bonds to increase their stability. Here, poly(vinylidene fluoride) (PVDF) was chosen to prepare a platform, which is insoluble in water, and polyethylenimine (PEI) was used to add amine groups on the surface of ENs to bind biological molecules via covalent conjugation. So, PVDF-PEI nanofibers were prepared on a glassy carbon electrode to immobilize an antimethamphetamine antibody (Anti-METH) as a model biomolecule. The obtained PVDF-PEI/Anti-METH was used for the bioelectrochemical detection of methamphetamine (METH), a common illicit drug. Bioelectrochemical detection of METH on PVDF-PEI/Anti-METH-coated electrodes was carried out by voltammetry in the range of 2.0–50 ng/mL METH. Moreover, the effect of dansyl chloride (DNC) derivatization of METH on the sensitivity of PVDF-PEI/Anti-METH was tested. Finally, METH analysis was carried out in synthetic body fluids. The obtained results showed that PVDF-PEI ENs can be adopted as an immobilization matrix for the biorecognition elements of biobased detection systems, and the derivative of METH (METH-DNC) increased the sensitivity of PVDF-PEI/Anti-METH.

## Introduction

Affinity sensors are a class of biosensors that are categorized according to their biorecognition principle.^1^ Affinity molecules such as antibodies, bioreceptors, nucleic acids, and aptamers are utilized as biological molecules for the design of affinity biosensors.^2^ Their basic principle is to follow affinity interactions between the biomolecule and its target. Among them, immunosensors are still popular ones for analyzing various size targets from small to big molecules.^3^ Novel materials and fabrication techniques provide the development of immunosensors with better performance characteristics. To enhance the surface properties of biomolecule-covered surfaces, beneficial immobilization matrices are crucial in the design of sensing interfaces.^4,5^ To fabricate immunosensors with high-quality sensing platforms, various materials such as natural^6^ and synthetic polymers^7^ and nanostructures such as gold nanoparticles,^8^ quantum dots,^9^ and carbon nanotubes^10^ have been used as a support matrix for the immobilization of antibodies. Also, electrospun nanofibers (ENs) in immunosensor design have been attracting great attention nowadays because of their unique properties.^11,12^ ENs may create a porous membrane structure with a high surface-to-volume ratio, which increases performance in a variety of applications.^13,14^ The nanofiber structure, characteristics, and morphology are required for a variety of applications.^15^ Electrospinning technology is mostly focused on synthetic polymers such as poly(ethylene oxide) (PEO),^16^ nylon,^17^ polyimide,^18^ and polyethylenimine (PEI).^19^ Among them, poly(vinylidene fluoride) (PVDF) is an electroactive polymer with unique properties such as electroactivity, chemical stability, and biocompatibility, making it suitable for a variety of applications including biosensors.^20^ PVDF has a high hydrophobicity; thus, it is not useful for the conjugation of biomolecules in aqueous solutions. To increase the hydrophilicity of PVDF and obtain chemical groups, PVDF nanocomposites have been prepared using various chemical agents such as MXene,^21^ graphene oxide,^22^ hydrophilic cellulose nanocrystal,^23^ etc. PEI is a good alternative to decrease the hydrophobicity of PVDF, and the presence of primary amines on PEI provides the covalent bond formation between biomolecules and PEI during the immobilization procedure of biological molecules.^24,25^ The numerous amine groups of PEI in the repeating units provide multipoint attachment of biomolecules via covalent bond formation. Also, its primary amines arrange for a positive charge on the surfaces and produce ionic interactions during the immobilization process.^26^

Methamphetamine (METH) is an illegal substance that causes tremendous exhilaration by stimulating the brain’s catecholamine neurotransmitter system.^27^ METH is more misleading and destructive than heroin, cocaine, and other typical illicit psychoactive chemicals, according to studies. It exerts far stronger mental control over misusers than physical control.^28^ In the past few decades, researchers have developed a variety of analytical methods to detect METH, including high-performance liquid chromatography-mass spectrometry (HPLC-MS),^29^ electrochemiluminescence (ECL),^30^ and gas chromatography-mass spectrometry (GC-MS).^31^ These methods are successful in the analysis of METH and other misused substances, but they are confined to the solution, and the analysis procedure is not only time-consuming but also expensive, requiring a large number of expensive instruments and a complex pretreatment and operation process. Because of their high accuracy, simple equipment, and easy operation, electrochemical biosensors are frequently utilized in target detection.^32^ Biosensors have some advantages such as low manufacturing cost, fast response time, usage of a small amount of target molecules for the analysis, high specificity and selectivity, rapid and continuous monitoring, and easy adaption to point-of-care test design.^33^

Dansyl chloride (DNC)-derivatized METH retained in a cartridge can then be subjected to further examination using systems with high selectivity and sensitivity. If this is possible, a series of screening and confirmation tests can be carried out following a single derivatization.^34^ DNC derivatization has some advantages in detecting METH such as appearing under ultraviolet (UV) light, specificity of DNC for METH (opiates, cocaine, and methylephedrine has not been derivatized with DNC), and the reduced interfering effect of some species in biological fluids.^34,35^ So, METH-DNC has been widely used in fluorescence sensing and HPLC applications.^34^ But its applicability to METH confirmation and determination has not been reported in a bioelectrochemical way.

In this study, PVDF was chosen as an ideal platform to prepare ENs to detect METH. PEI was utilized to add amine groups to the surface of ENs to covalently conjugate biological substances. To immobilize anti-METH, PVDF-PEI nanofibers were produced on a glassy carbon electrode. So, the bioelectrochemical detection of METH was carried out on a PVDF-PEI/Anti-METH-coated electrode by voltammetry, and the derivatization of METH (METH-DNC) increased the sensitivity of the bioelectrochemical detector.

## Experimental Section

### Materials

Methamphetamine (METH) was obtained from Cerilliant. The METH antibody (Anti-METH) was purchased from Arista Biological. Poly(vinylidene fluoride) (PVDF; average molecular weight: 534,000), polyethylenimine (PEI, branched, analytical standard, 50% (w/v) in H_2_O), TritonTM X-100, potassium hexacyanoferrate(III) [K_3_Fe(CN)_6_], 1-ethyl-3-(3-dimethylaminopropyl)carbodiimide (EDC), *N*-hydroxysuccinimide (NHS), bovine serum albumin (BSA; lyophilized; ≥96%), urea, uric acid, lactic acid, MgCl_2_, sodium carbonate (Na_2_CO_3_), and NH_4_Cl were obtained from Sigma. Dimethylformamide (DMF; 99.8%), acetone (Ac), and CaCl_2_ were obtained from Merck. Dansyl chloride (DNC; 98%) was purchased from PanReac AppliChem. All aqueous solutions were prepared in water from Millipore Milli-Q Plus. Artificial tear (Refresh) was purchased from the local pharmacy. Artificial sweat was prepared according to previous studies as follows:^36^ urea (CH_4_N_2_O): 22.0 mM; lactic acid (C_3_H_6_O_3_): 5.5 mM; NH_4_Cl: 3.0 mM; NaH_2_PO_4_: 100.0 mM; K_2_HPO_4_: 10.0 mM; CaCl_2_: 0.4 mM; MgCl_2_: 50.0 μM, and uric acid (C_5_H_4_N_4_O_3_): 25.0 μM.

### Apparatus

To obtain PVDF-PEI electrospun nanofibers, NanoWeb Electrospin 103 (MaviTech, Turkey) was used as a homemade electrospinning instrument. Surface images of PVDF-PEI nanofibers were taken by scanning electron microscopy (SEM; Carl Zeiss 300 VP) to show the morphological structure. SEM images were obtained by coating PVDF-PEI with gold for 90 s. The contact angles of PVDF-PEI nanofibers were measured with an Attention Theta Goniometer device. In the analysis performed using a goniometer, distilled water was adjusted to 5.0 μL and dropped onto the PVDF-PEI. A PalmSens potentiostat (Palm Instruments Houten, The Netherlands) was used for electrochemical measurements with three electrode configurations: a glassy carbon (GC) electrode as a working electrode, Pt as an auxiliary electrode (BASI, West Lafayette Indiana), and Ag/AgCl (Metrohm, Switzerland) as a reference electrode. The specific surface area of the PVDF-PEI film and PVDF-PEI ENs were obtained by the Brunauer–Emmett–Teller (BET) method using adsorption data.

### Electrospinning Conditions for the Preparation of PVDF-PEI ENs

PVDF with a final concentration of 15% (w/v) was dissolved in DMF/Ac (2:8; v/v) at 50 °C for 1 h. 15% PEI (w/v) was prepared by dilution in DMF and allowed to dissolve for 1 h. The prepared two separate polymer solutions of 15% PVDF and 15% PEI were mixed at a ratio of 9:1 (v/v) and stirred magnetically for 2 h at room temperature. Then, 1.75 × 10^–2^ M Triton-X100 was added to the mixture of hydrophobic (PVDF) and hydrophilic (PEI) polymers to secure homogeneity.^37^ The mixed polymer solution, which was prepared for electrospinning, was placed in a syringe with a needle diameter of 0.8 mm in a 2 mL volume and then placed in the syringe pump (ATABA AC–DC Adapter AT-511). Electrospinning parameters were determined after the required optimization studies. The optimized parameters were as follows: application voltage was 13–18 kV, the solution flow rate was 0.9–2.0 mL/h, and the distance between the syringe tip and collector plate was 12–15 cm. Electrospinning was carried out at 23–27 °C and 50–60% humidity. A GC electrode was used as a collector to obtain PVDF-PEI ENs. The PVDF-PEI modified GC electrode was left to dry and to evaporate the residual solvents at room temperature overnight.

### Anti-METH Coating on PVDF-PEI ENs

First, cleaning of the GC electrode was carried out to create a sensitive immunosensor. The GC electrode to be used as the working electrode was cleaned with alumina solutions with particle sizes of 0.05–0.1 and 1.0 μm. Afterward, the cleaning process was completed by leaving it in an ultrasonic bath for 1 min in a mixture of ethanol and distilled water (1:1; v/v). PVDF-PEI nanofibers were deposited on the GC electrode surface by electrospinning, taking into account the parameters determined during optimization. PVDF-PEI on GC was allowed to dry overnight. Anti-METH was diluted with 50 mM phosphate-buffered saline (PBS) (pH 7.4) to a final concentration of 2.5 μg/mL. Then, 0.2 M EDC and 0.4 M NHS were prepared in 50 mM PBS (pH 7.4) and mixed in a ratio of Anti-METH/EDC/NHS (1:1:2; v/v/v) to conjugate Anti-METH via covalent bonds onto PVDF-PEI. Overall, 20 μL of the mixture (Anti-METH/EDC/NHS) was dropped onto the PVDF-PEI nanofiber-modified GC surface and allowed to bind antibodies for approximately 2 h at room temperature.

### DNC Derivatization of METH

Dansyl chloride (DNC) (1.0 mM) was dissolved in Ac and 10.0 mM Na_2_CO_3_ was dissolved in distilled water and they were mixed at a ratio of 1:1 (v/v).^34^ The METH standard solution was diluted in methanol and added to the prepared DNC-Na_2_CO_3_ mixture at a ratio of 1:4 (v/v), and incubation was provided for 1 h at 45 °C. Thus, the METH-DNC derivative was realized.^34^

### Bioelectrochemical Detection of METH by PVDF-PEI/Anti-METH on the GC Electrode

**Scheme 1:**
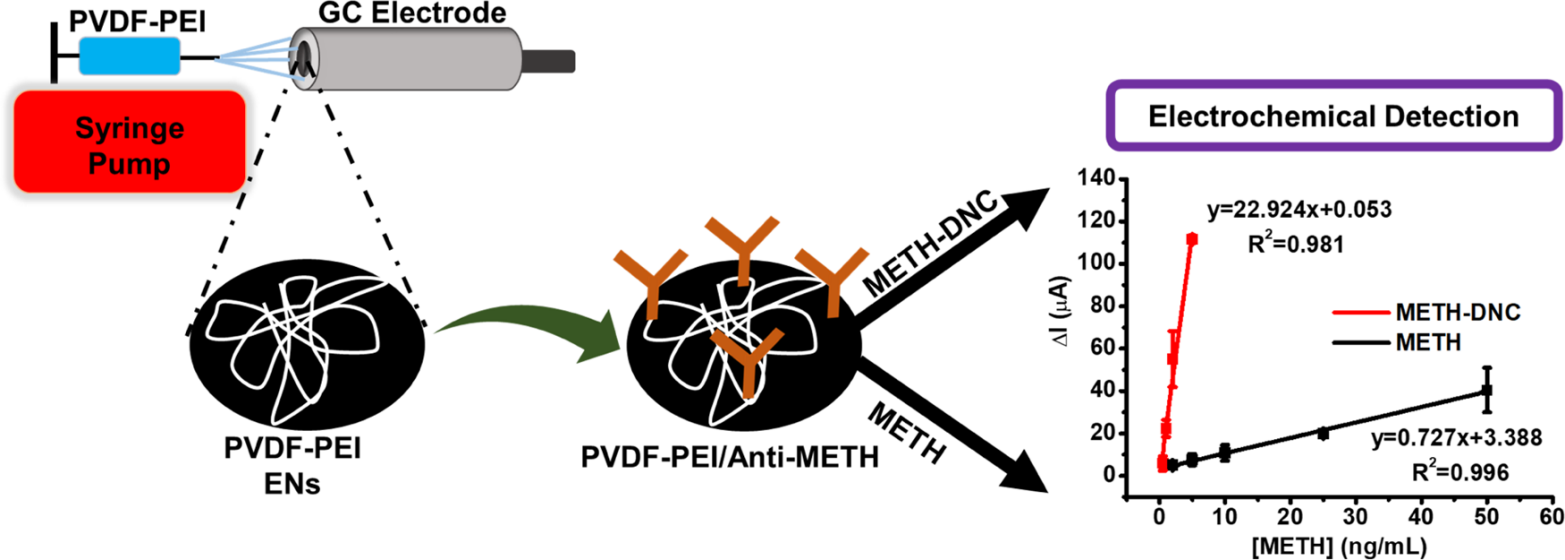
Schematic Representation of the PVDF-PEI EN Fabrication, Conjugation of Anti-METH, and Sensor Design

Differential pulse voltammetry (DPV), cyclic voltammetry (CV), and electrochemical impedance spectroscopy (EIS) measurements were performed on GC electrodes modified with PVDF-PEI and PVDF-PEI/Anti-METH to monitor the modifications step-by-step. Electrochemical experiments were carried out in 50 mM PBS (pH 7.4) with 50 mM K_3_[Fe(CN)_6_] and 0.1 M KCl.^38,39^ CV and DPV were performed in the potential range from −0.4 to +0.8 V, the frequency range was 0.21 × 10^–4^ – 100 kHz, the excitation voltage was 0.18 V, and dc potential was 10 mV for EIS measurements. Overall, 10 μL of METH or METH-DNC was dropped on the PVDF-PEI/Anti-METH-modified GC electrode surface and left for approximately 30 min for antibody interaction with METH. Measurements were taken after washing the surfaces with buffer at the same conditions. The representation for fabrication of PVDF-PEI ENs, conjugation of Anti-METH, and sensor design is shown in Scheme 1.

## Results and Discussion

### Characterization of PVDF-PEI ENs

**Figure 1:**
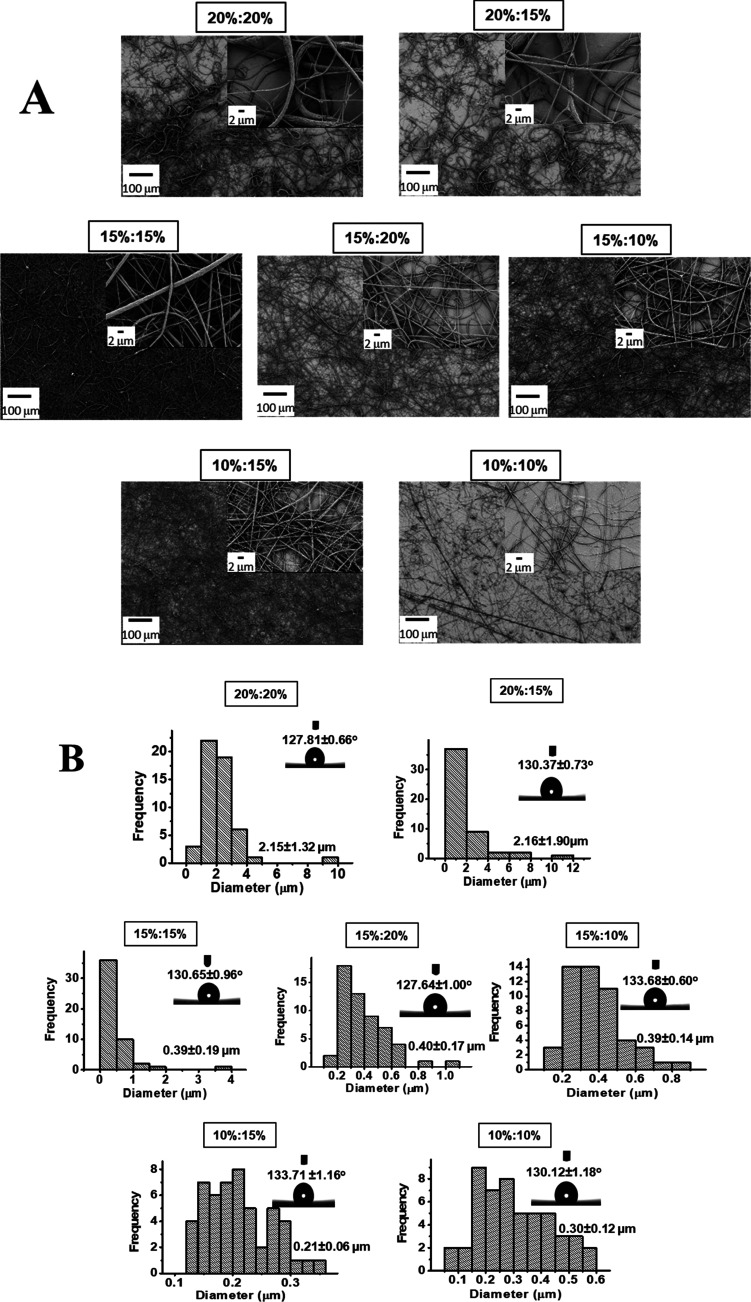
(A) SEM images of PVDF-PEI ENs, which are prepared using various concentrations of PVDF and PEI solutions (PVDF/PEI mixture in a 9:1 ratio (v/v), PVDF in DMF/Ac (2:8; v/v), and PEI in DMF; insets show SEM images of PVDF/PEI ENs in different magnifications). (B) Histogram for diameter distribution of PVDF-PEI ENs, which are prepared using various concentrations of PVDF and PEI solutions (PVDF/PEI mixture in a 9:1 ratio (v/v), PVDF in DMF/Ac (2:8; v/v), and PEI in DMF; insets show water drop images of contact angle measurements on PVDF/PEI ENs).

The effect of PEI amount on the morphology of PVDF-PEI ENs was evaluated by comparing SEM images, the diameter of PVDF-PEI nanofibers, and the contact angles of the PVDF-PEI surfaces. First, PVDF-PEI ENs were prepared using various concentrations of polymer solutions, as described in Table S1. The obtained SEM images of PVDF-PEI ENs with a mixture of PVDF and PEI in a ratio of 9:1 (v/v) are shown in Figure 1A. In all cases, nonbeaded nanofibers were obtained according to SEM images. The concentrations of PVDF and PEI affect the size of PVDF-PEI nanofibers.

A contact angle is created when a drop of fluid comes into contact with a flat solid surface, and the shape of the drop is determined by the relative magnitudes of the molecular forces that exist both inside the liquid (cohesive) and between the liquid and the solid surface (adhesive).^40^ The samples were sliced into thin strips and droplets of distilled water were administered to them gradually. Each water droplet has an exact volume of 3 μL. After placing the droplet on the sample, it was given 10 s to settle. This precise time was determined owing to the camera’s continual image of the computer program. The contact angle of a droplet was measured after this time. The findings can be classified as hydrophilic if the angle is less than 90°, hydrophobic if the angle is greater than 90°, very hydrophobic if the angle is greater than 120°, or superhydrophobic if the angle is greater than 150°. Figure 1B displays the histogram for the diameter distribution of PVDF-PEI ENs. The diameters of PVDF-PEI ENs significantly decrease with a reduction in PVDF and PEI concentration. The hydrophobic nature of PVDF is widely acknowledged.^4^ The contact angles (in 10 s) of PVDF ENs were unaffected by the PEI addition to their structure. After following the contact angle of PVDF-PEI in 15 min, it measured as 75.82°. All of these findings led to the selection of PVDF-PEI nanofibers with a larger distribution in a smaller diameter. For further studies, 15% (w/v) of PVDF and 15% (w/v) of PEI were used to form PVDF-PEI nanofibers mixing PVDF/PEI in a 9:1 ratio (v/v). By reviewing the literature, it was decided that the polymer mixtures were in appropriate ratios for the applications. For example, Shehata et al. fabricated nanocomposite membranes from PVDF and thermoplastic polyurethane (TPU) at blending ratios ranging from 9.5:0.5 to 7.0:3.0, showing enhanced mechanical properties.^41^ Trevino et al. studied the development and characterization of PVDF-conjugated polymer nanofiber-based systems. PVDF was blended with conductive polymers such as polyaniline, polypyrrole, polyindole, polyanthranilic acid, and polycarbazole. The highest piezoelectric performance was obtained from PVDF–polypyrrole nanofibers.^42^ In another study, PVDF ENs with copolymers of PMMA, by controlling the weight ratios from 9.5:0.5 to 8.0:2.0, were used for protein adsorption, water filtration, and oil–water separation.^43^ Pisarenko et al. performed contact angle measurements of PVDF fibers with 10 droplets for one type of sample. They investigated the effect of the collector speed on the contact angle and reported that the fibers electrospun at a low speed are more hydrophilic with a contact angle of 103.4 ± 4.2° than the electrospun nanofiber (131.8 ± 2.9°) at a high speed. The increased contact angle of PVDF fibers spun at a higher speed is attributed to the increased surface porosity, surface roughness, or parallel fiber alignment.^40^

**Figure 2:**
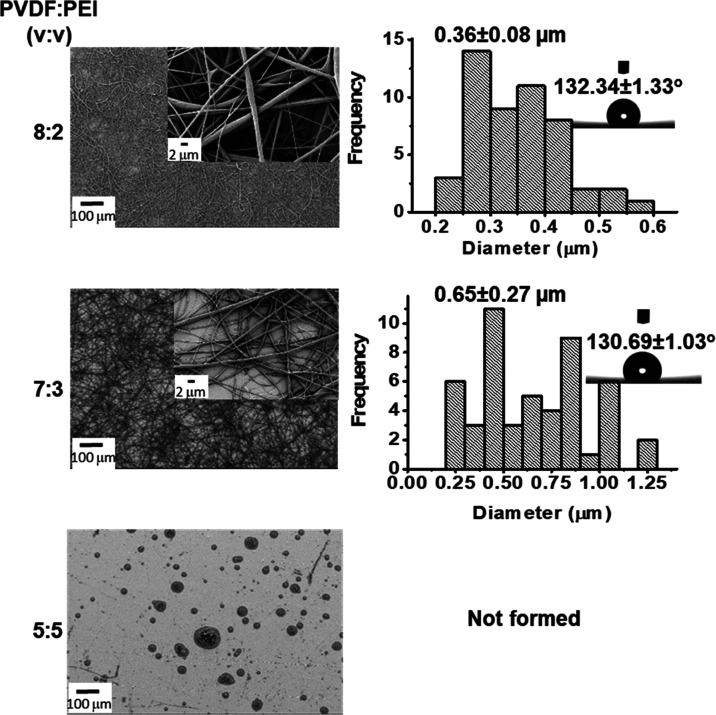
SEM images, the histogram for diameter distribution, and contact angles of PVDF-PEI ENs for different volume ratios (v/v) of PVDF and PEI solutions (15 wt % PVDF solution in DMF/Ac (2:8; v/v) and 15% (w/v) PEI in DMF).

In the following step, PVDF-PEI nanofibers were obtained at polymer solution ratios of 8:2, 7:3, and 5:5 (v/v, PVDF/PEI), keeping the concentration of the polymers at 15% (w/v). Figure 2 shows the SEM images, the histogram of fiber diameter distribution, and the contact angles of the PVDF-PEI ENs. It was determined that the homogeneous and thinner PVDF-PEI nanofibers with a narrower diameter distribution were obtained at a polymer ratio of 8:2 (v/v, PVDF/PEI). The contact angles of PVDF-PEI ENs were 132.34 ± 1.33 and 130.69 ± 1.03° for the volume ratios of 8:2 and 7:3, respectively. In our study, PVDF-PEI nanofibers were obtained by combining PVDF and PEI in a 9:1 (v/v) ratio with a solution in DMF/Ac (2:8), as shown in SEM images.

**Figure 3:**
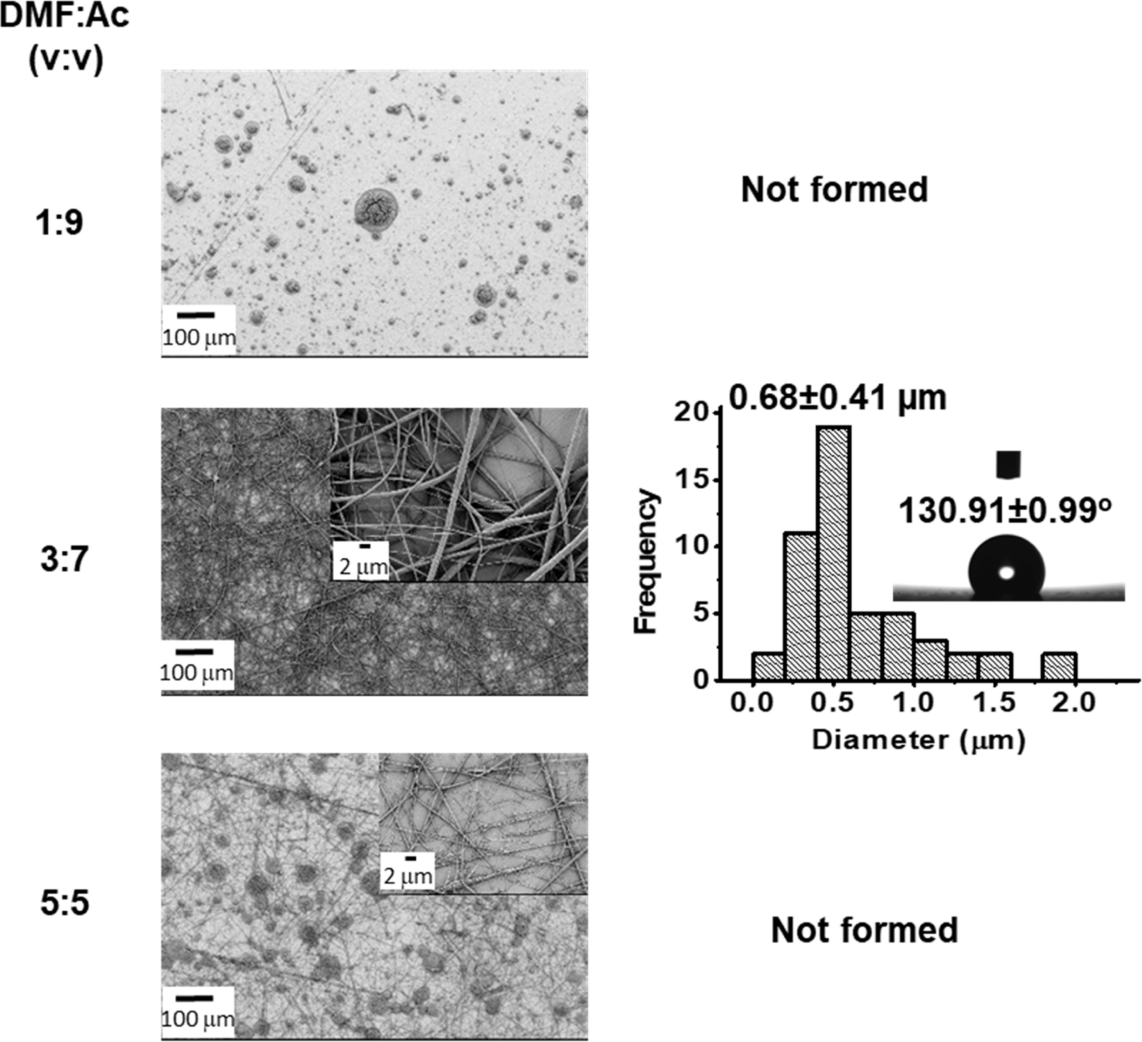
SEM images, the histogram for diameter distribution, and contact angles of PVDF-PEI ENs for different solvent ratios of DMF/Ac (15% (w/v) PVDF solution in DMF/Ac and 15% (w/v) PEI in DMF; PVDF/PEI ratio is 8:2 (v/v)).

The common solvents used in PVDF-based fibers are DMF, Ac, dimethylacetamide (DMAc), tetrahydrofuran (THF), and their mixtures.^44^ For instance, PVDF was dissolved in DMF/Ac at a volume ratio of 6:4.^45^ Fu et al. dispersed the PVDF/Zn(Ac)_2_ mixture in DMF/Ac with a mass ratio of 3:1. They found that the average diameter of PVDF/Zn(Ac)_2_ ENs without heat treatment was 508.60 ± 132.16 nm, while the average diameter decreased to 371.60 ± 84.23 nm after heat treatment. This decrease was associated with the conversion of zinc acetate into zinc oxide particles and the removal of PVDF.^46^ Mansouri et al. dissolved PVDF in a solution of DMF and THF with different ratios. They obtained an average diameter of less than 100 nm.^47^ Pisarenko et al. fabricated PVDF nanofibers in a DMSO/Ac solvent mixture in a 7:3 volume ratio. They found the average diameter of PVDF fibers collected at 300 and 2000 rpm as 966 ± 44 and 395 ± 13 nm, respectively.^40^ In another study, the diameter of the pristine PVDF nanofibers was stated as 76 ± 32 nm using DMF/water as a solvent.^48^ Sanchez et al. fabricated PVDF nanofibers with a diameter of 320 ± 115 nm using a PVDF/DMF solution at 15% (w/v).^49^ In this part, the effect of solvent ratios (DMF/Ac) used in a PVDF-PEI solution on the morphology of nanofibers was examined. Figure 3 demonstrates the electrospinnability of the PVDF-PEI solution at different solvent ratios. PVDF was dissolved in DMF/Ac at a volume ratio of 1:9, 3:7, and 5:5 (v/v), and PVDF-PEI nanofibers were prepared using 15% PEI in DMF [PVDF/PEI ratio was 8:2 (v/v)]. Nonbeaded and homogeneous nanofibers of PVDF-PEI were formed using DMF/Ac in a ratio of 3:7 (v/v) (Figure 3).

**Figure 4:**
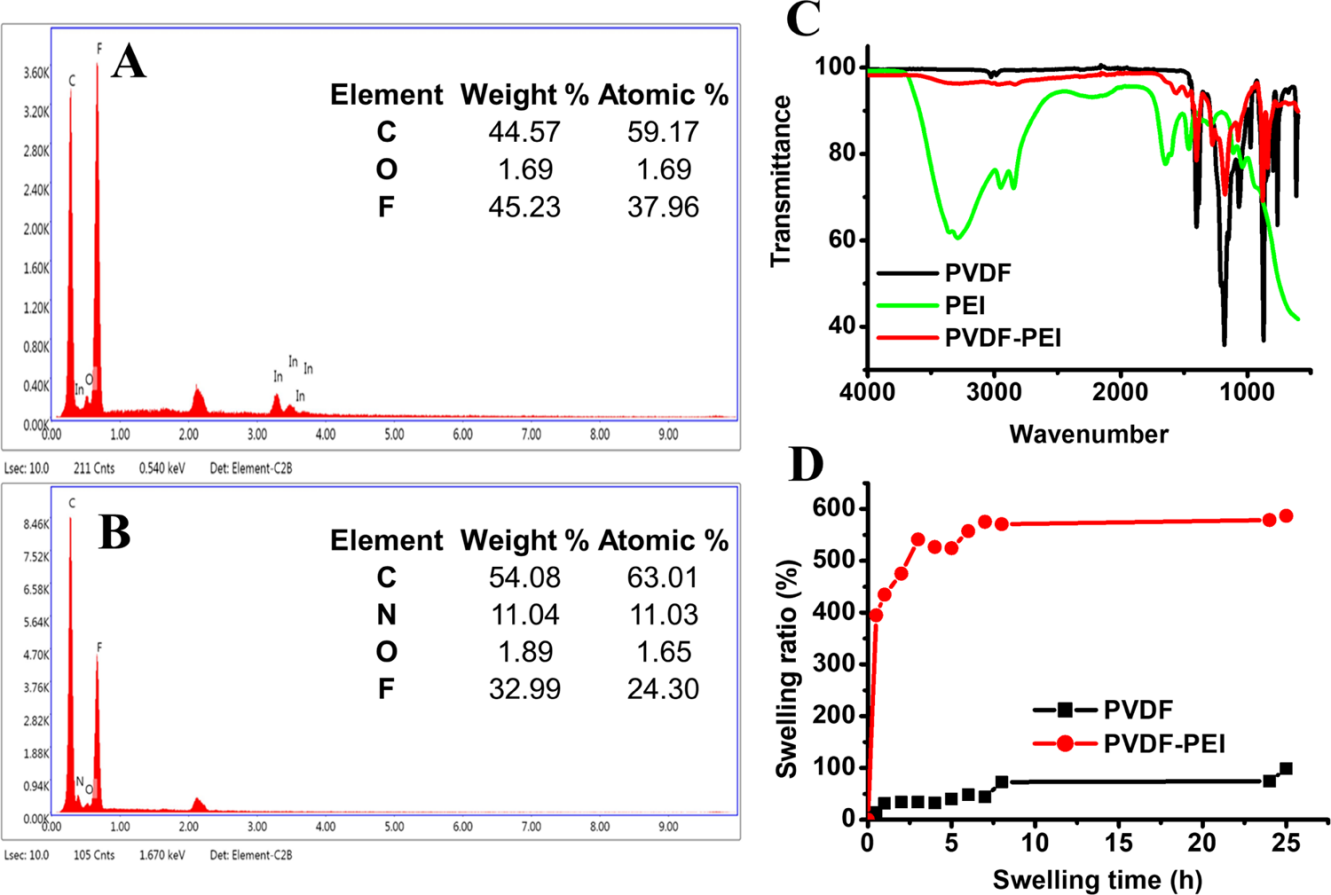
EDX for (A) PVDF and (B) PVDF-PEI; (C) FTIR spectrum of PVDF, PEI, and PVDF-PEI ENs; and (D) swelling ratios of PVDF ENs and PVDF-PEI ENs.

Energy-dispersive X-ray (EDX) spectroscopy was also used to confirm the presence of PEI in the blend fibers. The signals from carbon (0.277 keV) and fluorine (0.677 keV) were observed from PVDF (Figure 4A). The additional signal from nitrogen at 0.392 keV (Figure 4B) corresponded to the PEI. PVDF, PEI, and PVDF-PEI ENs are characterized using Fourier transform infrared (FTIR), and the obtained result is depicted in Figure 4C. The band at 490 cm^–1^ attributed to bending and wagging vibrations of the CF_2_ group results from the α phase of PVDF. At 840 and 745 cm^–1^, two distinctive bands from the β phase are assigned mixed modes of CH_2_/CF_2_ stretching vibrations. The stretching and bending vibrations of CH_2_ are found at 3016 cm^–1^ (ν_a_ CH_2_), 2978 cm^–1^ (ν_s_ CH_2_), and 1435 cm^–1^ (δ_s_ CH_2_), respectively.^50,51^ For PVDF-PEI ENs, the presence of N–H stretching may be pointed by the absorption band from primary/secondary amines at 3400 cm^–1^. Additionally, NH_2_ bending from primary amines appeared at 1655 cm^–1^.^52^ These bands are assigned to PEI embedded within the PVDF nanofibrous scaffold. The swelling ratio of PVDF and PVDF-PEI ENs is shown in Figure 4D. PVDF ENs have a maximum ratio of 578.87%, while PVDF-PEI ENs have 74.37%, indicating that the presence of PEI had a discernible impact on swelling at 24 h. The large increase in the swelling ratio could be ascribed to the hydrogen bonding between the water molecules and PEI. The BET surface area of composites for the PVDF-PEI film and PVDF-PEI ENs were found to be 1.07 and 2.22 m^2^/g, respectively. The adsorption average pore diameter (4 V/A by BET) of PVDF-PEI ENs was found to be 33.80 Å. It can be said that the formation of nanofibers instead of polymer films increased the surface area. The presence of pores on nanofibers provides the reaching of mediators onto the electrode surfaces.

**Figure 5:**
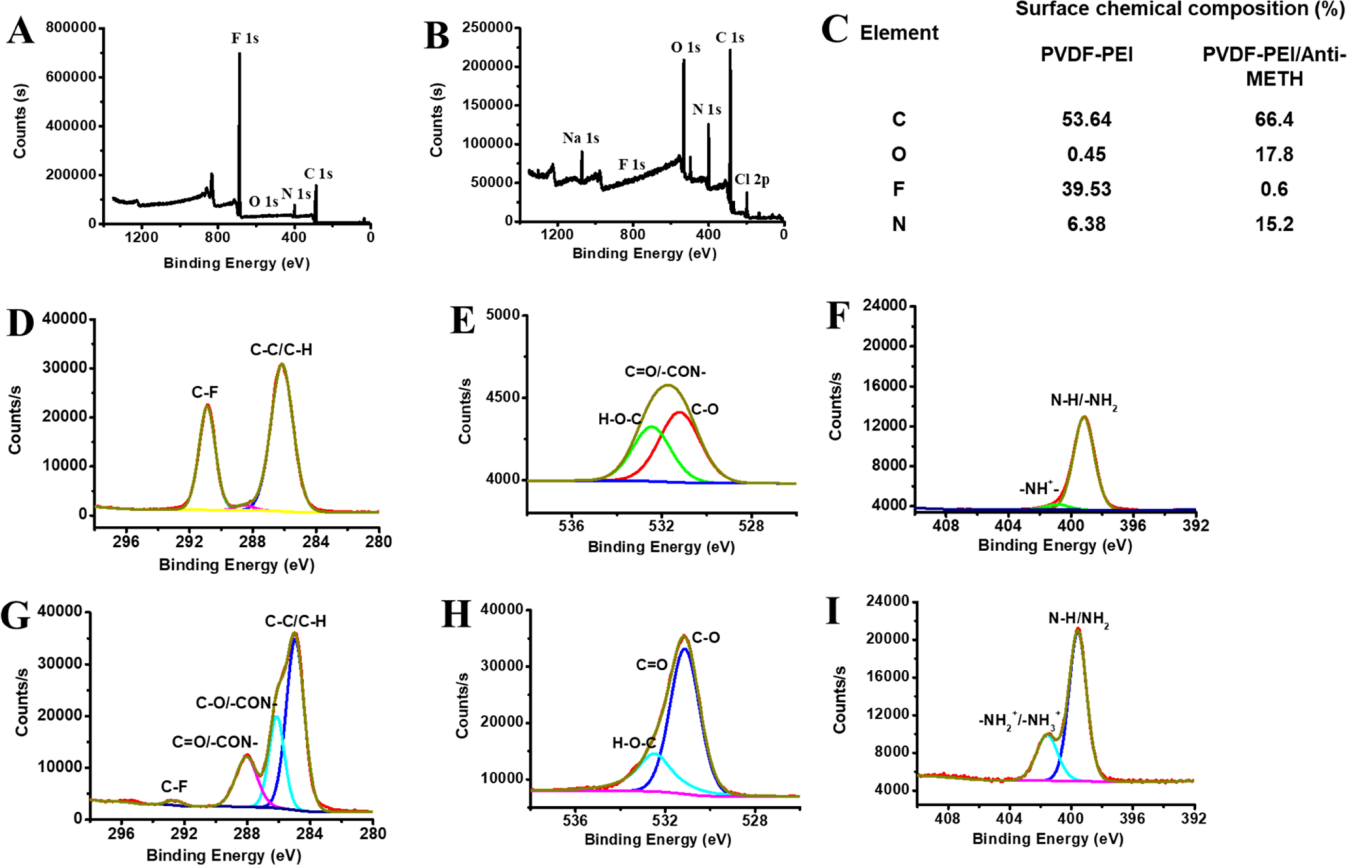
XPS survey spectrum of (A) PVDF-PEI ENs and (B) PVDF-PEI/Anti-MET; (C) table for percentage of the surface chemical composition of PVDF-PEI and PVDF-PEI/Anti-METH; (D) C 1s, (E) O 1s, and (F) N 1s spectra of PVDF-PEI; and (G) C 1s, (H) O 1s, and (I) N 1s spectra of PVDF-PEI/Anti-METH.

After demonstrating the fabrication of PVDF-PEI ENs, Anti-METH binding on them was carried out by covalent conjugation. The surface chemical composition of PVDF-PEI and PVDF-PEI/Anti-METH ENs was examined by X-ray photoelectron spectroscopy (XPS) measurements. XPS profiles of PVDF-PEI and PVDF-PEI/Anti-METH are shown in Figure 5, indicating the presence of the antibody on the nanofiber surface. Figure 5A,B displays the XPS survey of PVDF-PEI ENs and PVDF-PEI/Anti-METH, respectively. Figure 5C shows the chemical composition of PVDF-PEI ENs and PVDF-PEI/Anti-METH surfaces. C 1s, O 1s, and N 1s spectra of PVDF-PEI ENs are shown in Figure 5D–F, respectively. From deconvoluted C 1s spectra, the characteristic peaks of PVDF-PEI ENs at 286.16 eV (C–C/C–H), 288.54 eV (C=O/–CON), and 290.89 eV (C–F) are observed.^53,54^ Anti-METH conjugation on PVDF-PEI ENs results in a shift of the C=O bond from 288.54 to 288.05 eV (Figure 5G), indicating the formation of an amide bond between amines of PEI and the carboxyl group of antibodies. The amide linkage and electron delocalization on nitrogen were further corroborated by the change of the C–C peak from 286.16 to 285.55 eV. The amide bond formation between PEI and antibodies is therefore supported by the observed alterations in the C 1s, O 1s, and N 1s spectra (Figure 5G–I).

### Bioelectrochemical Detection of METH on PVDF-PEI/Anti-METH

**Figure 6:**
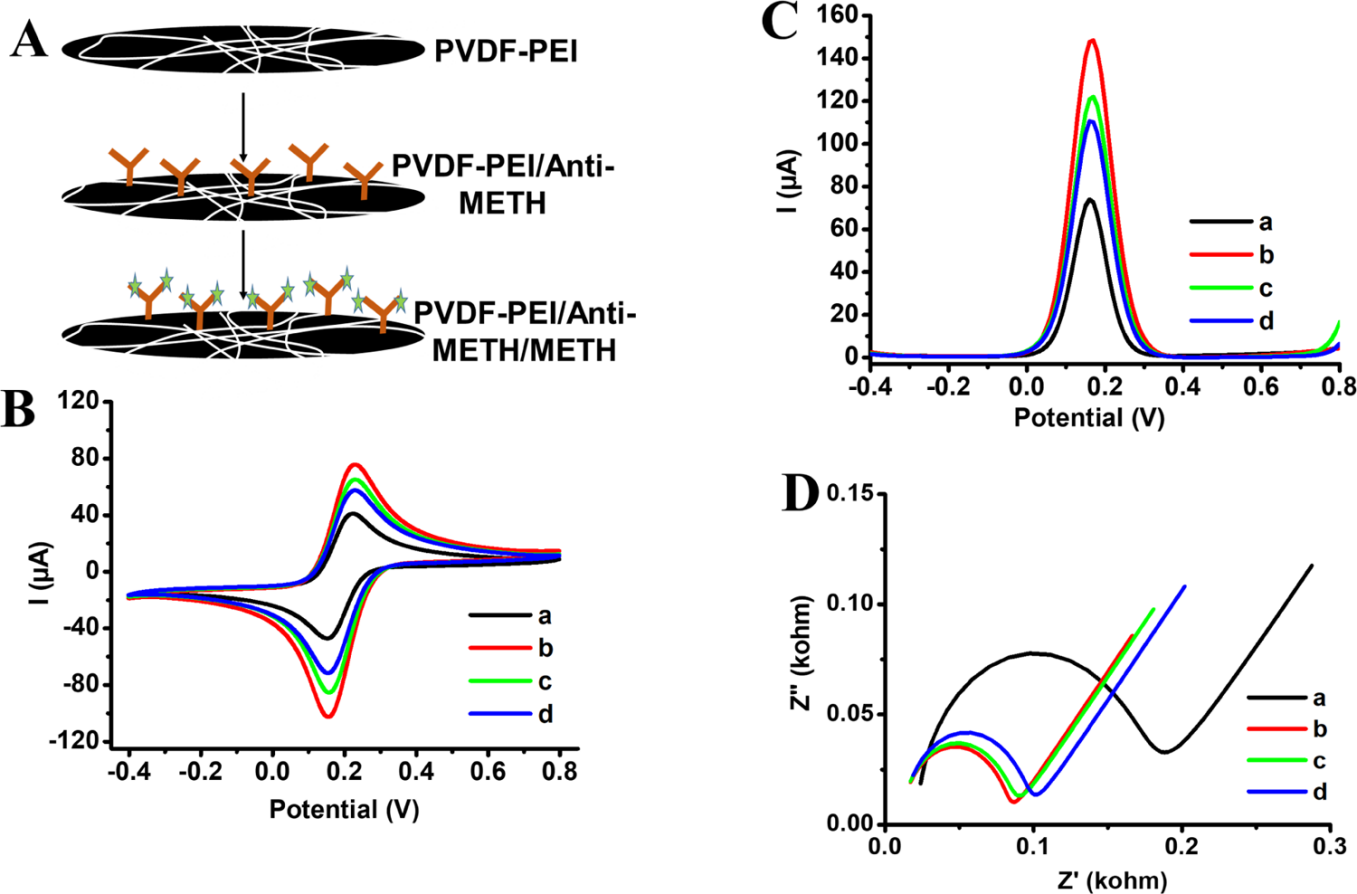
(A) Schematic for the preparation of PVDF-PEI/Anti-METH, (B) CV, (C) DPV, and (D) EIS of (a) bare, (b) PVDF-PEI, and (c) PVDF-PEI/Anti-METH-modified surfaces before and after the (d) addition of METH-DNC [in PBS (50 mM, pH 7.4), 5.0 mM [Fe(CN)_6_]^3–/4–^ and 0.1 M KCl were used during the measurement. The used METH concentration is 2.0 ng/mL].

After the characterization of PVDF-PEI/Anti-METH surfaces, electrochemical characterization was carried out. Figure 6A shows the preparation steps of PVDF-PEI/Anti-METH on the GC electrode. Figure 6B–D displays CV, DPV, and EIS profiles for the bare, PVDF-PEI ENs, and PVDF-PEI/Anti-METH-coated GC electrodes before and after the addition of METH, respectively. Potassium hexacyanoferrate(III) (HCF) was used as a redox mediator to follow the surface modifications of the GC electrode by PVDF-PEI and PVDF-PEI/Anti-METH. By applying CV and DPV methods, the redox peaks observed in the potential range from −0.4 to 0.8 V correspond to the oxidation and reduction peak of the HCF. The peak current of GC after modification by PVDF-PEI increased (Figure 6B(b),C(b)) because PVDF is a conductive material.^55^ After conjugation of Anti-METH, peak currents decreased (Figure 6B(c),C(c)) because the limitation barrier was formed for mediator transport. Then, METH was added as a target analyte and the peak currents decreased proportionally to its concentration (Figure 6B(d),C(d)). Figure 6D displays Nyquist spectra of bare, PVDF-PEI, and PVDF-PEI/Anti-METH-modified GC electrodes. The increase in the diameter of the semicircle provided the covering of GC surfaces, and it supported the data taken from CV and DPV measurements.

**Figure 7:**
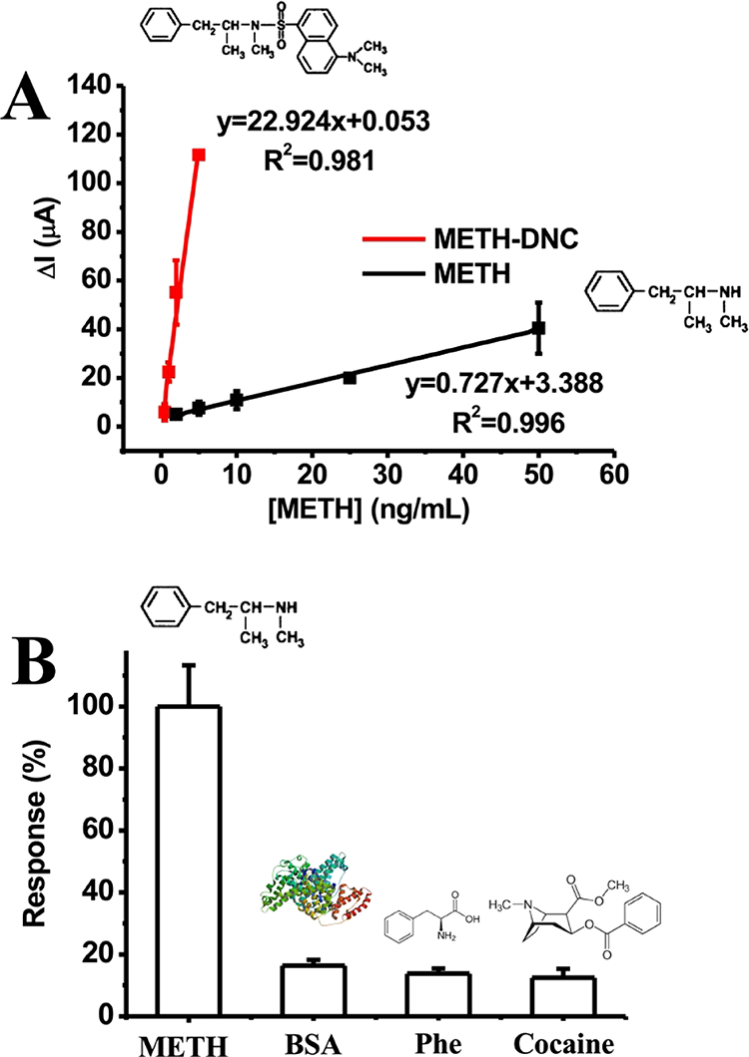
(A) Linear range for METH before and after DNC derivatization using the DPV method. (B) Specificity of PVDF-PEI/Anti-METH against METH [in PBS (50 mM, pH 7.4) error bars shows ± S.D.].

Linear ranges for METH detection were obtained using DPV measurements. Figure 7A shows the linear range curves obtained for METH standards at concentrations from 2.0 to 50 ng/mL in PBS with the corresponding equations: *I*(μA) = 0.727[METH] + 3.388 (*R*^2^ = 0.996) and METH-DNC standards at concentrations from 0.5 to 5.0 ng/mL in PBS with the corresponding equations: *I*(μA) = 22.924[METH] + 0.053 (*R*^2^ = 0.981). Under optimal conditions, the results displayed that the current signal enhanced with derivatization of the METH, resulting from the cumulative hindrance of the electrode surfaces. The limit of detection (LOD) and limit of quantification (LOQ) were calculated as 0.007 ng/mL (*n* = 6) and 0.024 ng/mL (*n* = 6) for METH (after DNC derivatization) using formula (3 SD/m for LQD and 10 SD/m) (SD: standard deviation and *n*: slope of the METH calibration curve), respectively.^56,57^ The PVDF-PEI/Anti-METH was more sensitive to METH than assays in the literature, which is shown in Table 1. SD and the coefficient of variation (cv) are used to indicate the reproducibility of bioanalytical systems. For this purpose, five consecutive measurements on different days were documented with 2.0 ng/mL METH-DNC using freshly prepared PVDF-PEI/Anti-METH. According to these measured values, SD and cv were calculated as ±0.076 and 4.901%, respectively. Covalent conjugation of the Anti-METH onto the PVDF-PEI-coated GC electrode surface with EDC as a zero-length cross-linking agent delivers high reproducibility. As illustrated in Figure 7B, the selectivity of PVDF-PEI/Anti-METH was tested in the detection of different potential interfering molecules in PBS. The obtained results show no obvious response, indicating that PVDF-PEI/Anti-METH achieves specific detection for METH.

**Table 1:** Comparison of the Analytical Performance of METH Assays in the Literature^a^

sensing mode	material	biological molecule	linear range	LOD	refs
ECL	AuNPs/ITO/PET	anti-METH	2.81–90.0 ng/mL	0.241 ng/mL	(58)
COL	Au@Ag	DNA aptamer	0.5–200 nM	0.1 nM (14.9 ng/mL^–1^)	(59)
FRET	CoOOH and C–Ds	DNA aptamer	5.0–156 nM	1.0 nM	(60)
COL	Au@Ag	DNA aptamer	0.5–200 nM	0.5 mM	(61)
CHEM	PCFS + SA–B-HRP	antibody	1.5–300 ng/mL	0.5 ng/mL	(62)
OPT	SiO_2_@CQDs@ms-MIPs	-	5.0–250 μM	1.6 μM	(63)
SERS	AuNPs	aptamer	10 pM-10 nM	7.0 pM	(64)
FL	GQDs@MIP	-	5–50 μM	1.7 μg/L	(65)
FOPPR	AuNPs	anti-METH	1–1000 ng/mL	0.16 ng/mL	(66)
ELEC	PVDF-PEI ENs	anti-METH	0.5–5.0 ng/mL	0.007 ng/mL	this study (METH-DNC)

a ECL: Electrochemiluminescent; AuNPs/ITO/PET: gold nanoparticle--functioned indium tin oxide-coated poly(ethylene terephthalate); COL: colorimetric; Au@Ag: gold–silver nanoparticles; FRET: fluorescence resonance energy transfer; CoOOH: cobalt oxyhydroxide nanosheet; C–Ds: carbon dots; ELEC: electrochemistry; CHEM: chemiluminescense; PCFS: portable chemiluminescent fiber-based immunosensor; SA–B-HRP: competitive enzyme-linked immunoassay and biotin–streptavidin-mediated peroxidase nanocomposite; SiO_2_@CQDs@ms-MIPs: green CQDs and mesoporous structured imprinting microspheres; OPT: optical; SERS: surface-enhanced Raman spectroscopy; FL: fluorescence; GQDs@MIP: graphene quantum dots embedded within molecularly imprinted polymer; and FOPPR: fiber optic particle plasmon resonance.

The validity and practicability of PVDF-PEI/Anti-METH were judged by the detection of METH in artificial samples. The prepared sample solutions were used for METH monitoring electrochemically. The detailed synthetic sample preparation process is presented in the Experimental Section according to the literature. The standard addition method was used for the detection of METH in samples; overall, 2.0 ng/mL of METH was added to the two samples. After the calculation of concentrations, the data are shown in Table 2. According to the results in Table 2, since PVDF-PEI/Anti-METH showed an acceptable recovery rate (between 95–105%),^67,68^ it can be judged that the PVDF-PEI/Anti-METH immunosensor can be used as a novel detection method for the detection of METH. Tears and sweat are liquid samples for the noninvasive detection of METH.

**Table 2:** Recoveries of METH in Artificial Samples Using the PVDF-PEI/Anti-METH Immunosensor

artificial Sample	added (ng/mL)	found (ng/mL)	recovery %
tear	2.0	2.041 ± 0.066	102.05
sweat	2.0	2.033 ± 0.271	101.65

## Conclusions

A new diagnostic sensor response was confirmed as the bioelectrochemical detection of dansyl chloride-derivatized methamphetamine on antibody-coated electrospun nanofibers. The effect of dansyl chloride (DNC) derivatization of METH on the sensitivity of PVDF-PEI/Anti-METH was tested. METH analysis was carried out in synthetic body fluids. The LOD was found as 0.007 ng/mL. Compared with the traditional METH detection method, this work has high sensitivity, low detection limit, good stability, reproducibility, and quick response. The obtained results showed that PVDF-PEI nanofibers can be adopted as an immobilization matrix for the biorecognition elements of biobased detection systems, and the derivative of METH (METH-DNC) increased the sensitivity of bioelectrochemical detectors.

## Supporting Information Available

Table for detailed experimental methods of the preparation conditions in PVDF-PEI electrospun nanofibers using various concentrations of PVDF and PEI solutions (PDF)
