# Supplementary material for: Antibody-Conjugated Electrospun Nanofibers for Electrochemical Detection of Methamphetamine
Source: ACS Appl Mater Interfaces. 2023 May 15;15(20):24109–19. doi: 10.1021/acsami.3c02266 (PMC10214377; doi:10.1021/acsami.3c02266)
Supplement: Supplementary file 1 — am3c02266_si_001.pdf [file am3c02266_si_001.pdf]

Supporting Information

Antibody-conjugated electrospun nanofibers for electrochemical detection of methamphetamine

Gozde Atik, Nur Melis Kilic, Nesrin Horzum*, Dilek Odaci* and Suna Timur*

*Prof. Dr. Suna Timur, Ege University Faculty of Science Biochemistry Department; suna.timur@ege.edu.tr, sunatimur@yahoo.com

*Prof. Dr. Dilek Odaci, Ege University Faculty of Science Biochemistry Department; dilek.odaci.demirkol@ege.edu.tr, dilekodaci.od@gmail.com

*Assoc. of Prof. Dr. Nesrin Horzum, İzmir Katip Çelebi University Department of Engineering Sciences; nesrin.horzum.polat@ikcu.edu.tr, n.horzum@gmail.com

Table S1. Preparation conditions of PVDF-PEI electrospun nanofibers using various concentrations of PVDF and PEI solutions.

PVDF (%)	Solvents for PVDF	PEI (%)	Solvent for PEI	Ratio of PVDF:PEI (v:v)	Parameters for electrospinning (Applied potential; Flow rate; Distance from needle to target)
10	DMF:Ac (2:8; v:v)	10	DMF	9:1	15 kV; 1.8 mL/h; 12 cm
10	DMF:Ac (2:8; v:v)	15	DMF	9:1	16 kV; 1.8 mL/h; 13 cm
15	DMF:Ac (2:8; v:v)	10	DMF	9:1	18 kV; 2.0 mL/h; 12 cm
15	DMF:Ac (2:8; v:v)	15	DMF	9:1	18 kV; 2.0 mL/h; 12 cm
15	DMF:Ac (2:8; v:v)	20	DMF	9:1	17 kV; 2.0 mL/h; 15 cm
20	DMF:Ac (2:8; v:v)	15	DMF	9:1	17 kV; 2.0 mL/h; 15 cm
20	DMF:Ac (2:8; v:v)	20	DMF	9:1	16 kV; 2.0 mL/h; 13 cm
